# The Associations of Body Image Perception with Serum Resistin Levels in Highly Trained Adolescent Estonian Rhythmic Gymnasts

**DOI:** 10.3390/nu13093147

**Published:** 2021-09-09

**Authors:** Liina Remmel, Jaak Jürimäe, Anna-Liisa Tamm, Priit Purge, Vallo Tillmann

**Affiliations:** 1 Institute of Sport Sciences and Physiotherapy, Faculty of Medicine, University of Tartu, 51008 Tartu, Estonia; jaak.jurimae@ut.ee (J.J.); priit.purge@ut.ee (P.P.); 2 Department of Physiotherapy and Environmental Health, Tartu Health Care College, 50411 Tartu, Estonia; annaliisatamm@nooruse.ee; 3 Institute of Clinical Medicine, Faculty of Medicine, University of Tartu, 50406 Tartu, Estonia; vallo.tillmann@kliinikum.ee; 4 Institute of Clinical Medicine, Children’s Clinic of Tartu University Hospital, 50406 Tartu, Estonia

**Keywords:** rhythmic gymnasts, body image perception, resistin, body composition

## Abstract

Rhythmic gymnasts (RGs) are more likely to be dissatisfied with their body mass and shape compared to untrained controls (UCs). However, due to the lack of information, the aim of this study was to investigate the associations of body image perception (BIP) with body composition, daily energy consumption and different blood biochemical markers in adolescent RGs compared to UCs. Thirty-three highly trained RG girls and 20 UC girls aged 14–18 years participated in this cross-sectional study. Height, body mass, body composition, energy intake, resting energy expenditure, training volume and different blood biochemical markers were measured. The body attitude test (BAT) was used to evaluate the BIP of the participants. There were no differences in the total BAT scores between the groups. In RGs, the BAT score correlated positively with the serum resistin level (r = 0.35; *p* = 0.047). A stepwise multiple regression analysis showed that 40.8% of the variability in the BAT score was determined by resistin and BMI. The association of BIP with resistin values was observed only in RGs. In conclusion, our findings add to the increasing evidence that resistin may be a link between BIP and body composition, most likely through fat mass, in adolescent female RGs.

## 1. Introduction

High-level participation in different sport events demands not only a high physical ability, but also a good mental capability [1]. Mental problems are not rare in elite athletes, with anxiety and depression being the most common disorders [1]. In comparison to other athletes, rhythmic gymnasts (RGs) are more likely to be dissatisfied and concerned with their body mass and shape [2] as visual appearance is very important in this field. Body image perception (BIP) has been associated with obesity either as a cause or as a result that affects body mass control behaviours [3]. Although BIP misperception (either underestimation or overestimation) occurs more in obese individuals [3], it has been found that RGs have a negative self-perception of their body image and their body size [4]. An inadequate energy intake is common in elite RGs [5] and may lead to a negative energy balance in these athletes [6]. Abnormal eating habits are associated with permanent worries about their body mass and BIP [7] and this is particularly pronounced during adolescence—a time when RGs have high expectations for themselves and have an impaired BIP [8]. In addition, the accumulation of body fat and other pubertal changes causes their bodies to deviate from the prepubertal thin body shape considered as an ideal in this sport field, which can cause an impaired BIP among adolescent girls [9]. Given that RGs are concerned with their body appearance, it is important to measure their BIP and the modifiable risk factors during adolescence to prevent problems in later life.

The biochemical mechanism linking an impaired BIP and body composition is not clear. Adipokines, such as leptin, adiponectin and resistin might be possible candidates to characterise BIP by body composition variables, as positive correlations between these adipokines and BMI have been found [10]. In addition, a recent study in adolescent girls with anorexia nervosa found that the serum resistin concentration was associated with depressive symptoms [11]. Specifically, it has been suggested that resistin can inhibit dopamine and noradrenaline release in the hypothalamus and can decrease intrasynaptic monoamine levels, which in turn could lead to the predisposition of the development of depression symptoms [12]. Accordingly, this may suggest that resistin could be involved in the regulation of emotions and behaviours. We set up a study to describe body image perception (BIP) in highly trained adolescent RGs compared to UCs and the associations of BIP with body composition, daily energy consumption and different blood biochemical markers including resistin.

## 2. Methods

### 2.1. Participants

The participants of this study were 53 young female adolescents aged 14 to 18 years who were divided into two groups: rhythmic gymnasts (RGs; *n* = 33) and untrained controls (UCs; *n* = 20). All UCs were eumenorrheic females who reported to have regular menstrual cycles during the last six months. In the RG group, 22 girls were eumenorrheic and 11 girls had secondary amenorrhea. Menstruating athletes and UCs were examined during the follicular phase when blood samples were obtained between 7 and 11 days from the onset of menstruation [13]. The first testing session consisted of blood sampling, dietary assessment and the completion of questionnaires about BIP and training characteristics. The second testing session consisted of resting energy expenditure (REE) and body composition assessments. All subjects and their parents had to sign an informed consent. The Medical Ethics Committee of the University of Tartu, Estonia (ethical approval code number 274/T-3, date 16 October 2017) approved the current study.

### 2.2. Medical and Menstrual History

Information about the age at menarche, changes in menstrual cycle, possible oral contraceptive use, past and current illnesses, use of medications, vitamins and food supplements were collected [13]. Regular menstrual cycles were defined as menses occurring every 24–35 days [13]. Secondary amenorrhea was classified as the absence of at least three consecutive menstrual cycles after the initiation of menses, including no regular menses for at least six months [14].

### 2.3. Body Composition

The participants’ body height (Martin metal anthropometer) and body mass (A&D Instruments Ltd., Abingdon, UK) were measured to the nearest 0.1 cm and 0.05 kg, respectively. The calculation of the body mass index (BMI) was completed as a ratio of body mass to the height squared (kg/m^2^). The dual-energy X-ray absorptiometry (DXA) using the DPX-IQ densitometer (Lunar Corporation, Madison, WI, USA) equipped with proprietary software, version 3.6 was used to measure the body composition of the participants. All participants were scanned in light clothing. They were laid flat on their backs, arms on their sides and were motionless. Total body (TB) fat mass (FM; kg) and lean body mass (LBM; kg) were measured and the TB FM% was calculated. The same examiner conducted the DXA measurements and evaluated the results. The coefficients of variations (CVs) for the obtained results were less than 2%.

### 2.4. Dietary Assessment

Energy and nutrient intakes were assessed using the average of three 24 h dietary recalls. It had to include two weekdays (i.e., Thursday and Friday) and one weekend day (Saturday). All participants were instructed to keep their ordinary diet where they were allowed to eat as much as they ate usually during the study period but were asked to maintain their usual dietary habits and everyday physical activities as before the assessment [15]. To evaluate estimated portion sizes, cup and bowl sizes were used. The Nutridata System for Research (www.nutridata.ee; accessed on January 2018; National Institute for Health Development, Tallinn, Estonia) was used to analyze the results. The dietary analysis included energy, carbohydrates, proteins, fats, vitamin D, vitamin C, calcium, magnesium, potassium, iron and sodium. Total daily food intake was also included in the analysis. The daily energy and nutrient intakes were calculated as the average of the three days [15].

### 2.5. Resting Energy Expenditure

Resting energy expenditure (REE) was measured in the morning after an overnight fast. Participants were asked to avoid different kinds of intense physical activity for the 24 h period before the REE measurement. After voiding, the subjects laid down for 15 min before the measurement of oxygen consumption (VO_2_) and carbon dioxide production (VCO_2_) over a 30 min period. The first 5 min and last 5 min of the measurement were removed to obtain an adequate result [16]. A portable open circuit spirometry system (MetaMax 3B, Cortex Biophysic GmbH, Germany) was used, the data were recorded in 10 s intervals and the mean of the 20 min was used to calculate REE according to Weir’s equation (1949): Basal metabolic rate (BMR) (kcal/min) = 3.9 [VO_2_ (l/min)] + 1.1 [VCO_2_ (l/min)]; REE (kcal/day) = BMR × 1440 min [17].

### 2.6. Blood Analysis

A venous blood sample was collected between 8:00 and 9:00 a.m. from an antecubital vein. The blood serum was separated and then frozen at −80 °C for later analysis. Serum ferritin, insulin, leptin, interleukin-6 (IL-6), plasminogen activator inhibitor-1 (PAI-1), resistin and tumor necrosis factor-a (TNF-a) levels were measured using “Metabolic Syndrome Array I” kit by Evidence^®^ Biochip Technology (Randox Laboratories Ltd., Crumlin, UK). None of the metabolic biomarkers were below the detection limits. The intra-assay coefficient of variation (CV) was between 2 and 5% and the inter-assay CV was between 8.2 and 11.5% for all measured markers.

### 2.7. Body Attitude Test

During the first testing session, the subjects filled out a body attitude test (BAT) (Appendix A) to evaluate their body image perception (BIP) [18]. The BAT is intended for female subjects to measure their subjective body experience and their attitude towards their body as BIP. Specifically, the BAT consists of 20 items to be scored on a 6-point Likert scale from *never* to *always*. The higher the score, the more the body experience deviates. In addition to the total score, subscales of factor 1 (negative appreciation of body size), factor 2 (lack of familiarity with one’s own body), factor 3 (general body dissatisfaction) and the rest factor were calculated [18]. 

The cut-off point above 36 was used to identify subjects with a very high likelihood of having eating disorders and an impaired BIP [19].

### 2.8. Statistical Analysis

All statistical analyses were completed using SPSS softwere version 21.0 package for Windows (Chicago, IL, USA). Standard statistical methods were used to calculate the means and standard deviations (SD). The Shapiro–Wilk method was used to evaluate the normality of the data. Data that were not normally distributed were logarithmically transformed prior to the analysis to approximate a normal distribution. The differences between the BAT scores and the measured biomarkers were evaluated using an independent *t*-test. In addition, effect size (ES) was calculated as the pairwise comparison of quantitative variables and was considered to be small if ES > 0.1, moderate if ES > 0.3 or large if ES > 0.5. The odd ratio (OD) was calculated as a chi squared test for detecting any possible statistical differences of an impaired BIP. Spearman correlations were conducted to describe the relationships between the variables. A stepwise multiple regression analysis was performed to determine the independent effect of body height, body mass, BMI, body composition characteristics and different blood biochemical characteristics on the BAT scores. The significance level was set at *p* < 0.05.

## 3. Results

The studied RG and UC groups had similar body height, body mass and BMI values (Table 1). As expected, in the RGs the TB FM% and TB FM values were lower (*p* < 0.05) and the TB LBM was higher compared to the UCs. The mean energy intake and REE were similar between the groups (Table 1). In addition, insulin, leptin, IL-6 and PAI-1 levels were significantly lower in the RG compared to the UC group (Table 2).

There were no differences in the total BAT scores and its subscales between the RG and UC groups (Table 3). However, there were more girls (8/33; 24.2%) with an impaired BIP (BAT > 36) in the RG group compared to the UC group (3/20; 15%), but the difference was not statistically different. The RG and UC groups did not statistically differ (*p* = 1.000) in the frequency of impaired BIP and the OR = 1.07 [0.29–3.92]. When the RG and UC groups were combined, the serum resistin concentration was not different between the girls with a BAT > 36 and those with a BAT < 36.

**Figure 1:**
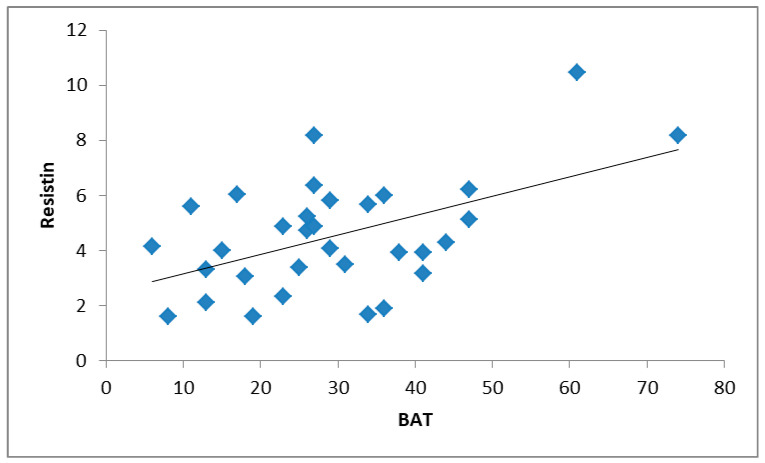
Correlation between resistin and BAT in RGs (r = 0.35; *p* = 0.047).

**Figure 2:**
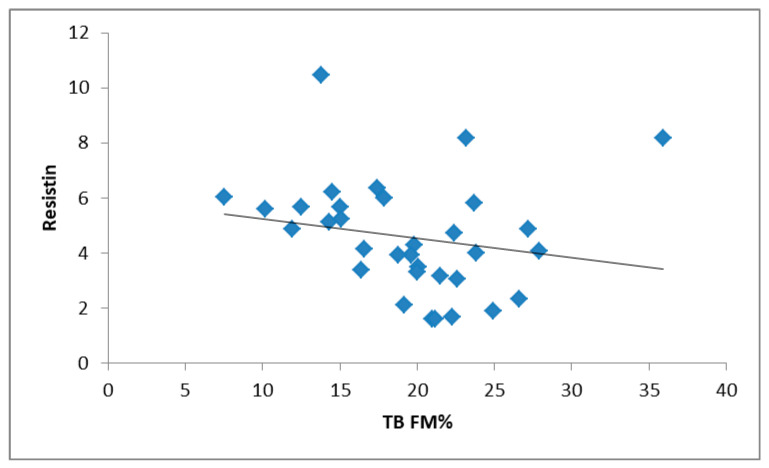
Correlation between resistin and TB FM % in RGs (r = −0.36; *p* = 0.040).

In the RG group, the BAT score correlated positively with the resistin level (r = 0.35; *p* = 0.047) (Table 4; Figure 1). In addition, the resistin level correlated negatively with TB FM% (r = −0.36; *p* = 0.040) (Figure 2) and positively with training duration (r = 0.37; *p* = 0.034). The stepwise multiple regression analysis showed that 40.8% of the variability of the BAT was determined by resistin and BMI (Table 5).

In the UC group, the BAT correlated with BMI (r = 0.48; *p* = 0.039). The stepwise multiple regression analysis showed that 21.0 % of the variability of the BAT was determined by total body mass (Table 5).

## 4. Discussion

This study found that nearly every fourth rhythmic gymnast (24.2%) had an impaired body image perception (BIP) compared to every seventh girl in the untrained control group (15%), but the difference was not statistically significant. The main finding of the current study is that the association of body image perception, measured by the body attitude test (BAT) with serum resistin values was observed in adolescent RGs. This finding suggests that serum resistin could be a link between BIP and body composition, most likely through fat mass.

It is known that young females competing in leanness and weight-class sports, such as RGs, are at a high risk of developing patterns of disordered eating, which is also associated with psychological problems in later life [20]. The prevalence of clinical eating disorders among Norwegian female elite high-intensity sport athletes was high—32.2% [21]. Nowadays, female athletes may become increasingly attractive in the media, which is affected by society and that may be the reason the beauty ideal has emphasised visual appearance as athletic and thin [22].

Therefore, it is very important to find the girls who are at risk of these health problems and possible risk factors. Body image perception is dependent on body mass and both over- and underweight individuals have been associated with an impaired BIP [23]. In our study, 21% of the variability of the BAT score in the control girls was determined by body mass. The mean body mass of the UC group (58.4 kg) represents the 50th centile on the Estonian adolescent girl’s growth chart [24] indicating that the prevalence of impaired BIP in the UC group (15%) could represent the whole population of Estonian girls at this age. Impaired BIP is not a disease itself, but a high BAT score, a measure of BIP, could suggest a high likelihood of eating disorders later in life [19]. In addition, an impaired BIP may predict unsafe weight loss behaviours among adolescent normal weight females; thereby, excessive high-volume training to change the body appearance is associated with impaired BIP [25].

Our results showed that in RGs 24.1% of the variability of the BAT score was determined by resistin alone and 40.8% was determined by resistin together with BMI, suggesting an important role of this biomarker in BIP. We also described that the serum resistin level was positively correlated to BIP measured by the BAT. Resistin, an adipose tissue-derived low-grade inflammation marker is produced by different cells: adipocytes, macrophages, myocytes and pancreatic cells. The physiological role of resistin in humans is still unclear and studies in animals have shown different and conflicting results [12]. For instance, obesity induced by a high-fat diet or the mutation of the leptin gene or receptor is associated with increased circulating resistin concentrations, whereas resistin expression was downregulated in rodent models with diet-induced obesity and suppressed by free fatty acids [12]. In our UC group we did not find significant correlations between the serum resistin level and body mass, BMI or TB FM%, similar to the study of 302 adolescents where the resistin level correlated with BMI, triceps skinfold, arm circumference, arm fat area and FM among obese subjects, but not in adolescents with a normal BMI [26]. However, in the RG group, the serum resistin level correlated negatively with TB FM%. Therefore, the association between resistin and the BAT score could be mediated through body composition, most likely through FM.

In the general adult population, the resistin level has been found to have a inverse association with physical activity [27]. Our study did not find differences in the serum resistin level between the RGs and the UCs, which is similar to the study by Roupas et al. [28]. However, the training duration was significantly correlated with the resistin level in the RG group but not in the UC group. It has been suggested that rhythmic gymnastics is more likely anaerobic and therefore the long-term training has no effect on the resistin level [28].

There have been many studies linking resistin with the development of depression [11,29,30]. There is some evidence that resistin can inhibit dopamine and noradrenaline release in the hypothalamus and can decrease intrasynaptic monoamine levels, which could lead to a predisposition toward the development of depression symptoms [12]. Resistin is decreased in patients with anorexia nervosa [31]. To the best of our knowledge, no studies have examined the association of BIP with resistin and depression in RGs. Klinkowski et al. [32] showed that even though RGs may look physically similar to girls with anorexia nervosa, from the psychopathological point of view, they have very different profiles. The self-report questionnaire about psychological symptoms showed that patients with anorexia nervosa scores were significantly higher on almost all scales and no parallels in psychopathology were found between elite RGs and anorexia nervosa patients. The RG group suffered less from psychological distress than the anorexia nervosa group [32]. It has been found that depression increases significantly during adolescence, particularly in girls [33]. The association between BIP and depressive behaviours is not clear, in particular whether an impaired BIP causes depressions or whether patients with depression are more sensitive or more likely to be dissatisfied with their body image [34]. Filho et al. [35] concluded that body image dissatisfaction was associated with symptoms of a depressive disorder only in those adolescents who were dissatisfied with their body image due to being overweight, but not in those adolescents who were dissatisfied due to thinness. Probably, adolescent girls that perceived themselves to be overweight but had a normal weight are more liable to develop symptoms of a depressive disorder compared to those that had a more adequate perception of their own body mass [36]. Unfortunately, our questionnaire did not consist of questions about depression. However, different studies support the finding that resistin may be a biochemical marker linking an impaired BIP, body composition and the development of depression [29,30]. Further studies are needed before any conclusions between serum resistin level and the development of depression in high intensity trained RGs can be made.

In RGs, the average energy intake was under the energy requirements for female athletes. It is known that the recommended minimum value of the daily energy intake for female athletes is between 1800 and 2000 kcal [37]. However, our results are similar to the study by Silva and Paiva [5], where the average daily energy intake was 1629 (kcal) in 16–18-year-old gymnasts. This finding indicates that the average daily energy intake of RGs is usually lower than the recommended figure.

To the best of our best knowledge, this is the first study investigating the associations between BIP and body composition, daily energy consumption and different blood biomarkers in RGs. In addition, the participants in this study were high level Estonian RGs from different sport clubs. These are the definite strengths of the study. However, there are also some limitations. We did not use a questionnaire about depression which would have given more details about the development of depression symptoms. In addition, the current study is cross-sectional and this limitation reduces the ability to make causal inferences.

## 5. Conclusions

Highly trained RGs did not have an impaired body image perception (BIP). The positive correlation between serum resistin level and the body attitude test as a measure of BIP, suggests that resistin may be a link between BIP and body composition in adolescent female RGs. However, further studies are needed to clarify the exact mechanism behind this association.

## Figures and Tables

**Table 1:** The main characteristics and fat parameter data in the rhythmic gymnasts (RGs) and untrained controls (UCs) female groups.

	RG (33)	UC (20)	*p* Value	ES
Age (yrs)	16.0 ± 1.2	16.5 ± 1.6	0.202	0.39
Body height (cm)	166.8 ± 5.3	166.8 ± 5.0	0.976	0.01
Body mass (kg)	55.7 ± 7.0	58.4 ± 7.4	0.180	0.42
BMI (kg/m^2^)	20.0 ± 2.0	21.0 ± 2.2	0.100	0.52
TB FM% (%)	19.6 ± 5.7 *	30.4 ± 6.2	<0.001	1.55
TB FM (kg)	11.2 ± 4.3 *	17.8 ± 4.8	<0.001	1.36
TB LBM (kg)	42.2 ± 4.1 *	37.7 ± 3.7	<0.001	1.06
Energy intake (kcal/day)	1644.2 ± 424.0	1571.7 ± 295.5	0.505	0.15
REE (kcal/day)	1495.0 ± 208.1	1520.3 ± 208.3	0.669	0.12
Menarcheal age (yrs)	13.6 ± 1.2 *	12.5 ± 0.7	<0.001	1.03
Training experience (yrs)	10.3 ± 0.9 *	-	-	-
Training duration (h/week)	17.6 ± 5.3 *	2.1 ± 1.3	<0.001	1.84

BMI, body mass index; TB FM, total body fat mass; TB LBM, total body lean body mass; REE, resting energy expenditure; ES, effect size. Data are described by means ± SD. Significant (*p* < 0.05) difference between RGs and UCs *. ES is small if ES > 0.1, moderate if ES > 0.3 or large if ES > 0.5.

**Table 2:** The blood biomarkers data in the RGs and UCs female groups.

	RG (33)	UC (20)	*p* Value	ES
Ferritin (ng/mL)	35.84 ± 14.53	24.68 ± 23.79	0.038	0.55
Insulin (ulU/mL)	6.12 ± 2.27 *	8.65 ± 3.54	0.002	0.90
Leptin (ng/mL)	1.23 ± 0.62 *	4.29 ± 3.40	<0.001	1.34
IL-6 (pg/mL)	0.79 ± 0.60 *	1.59 ± 1.23	0.003	0.59
PAI-1 (ng/mL)	17.85 ± 7.10 *	31.46 ± 13.61	<0.001	1.20
Resistin (ng/mL)	4.57 ± 2.02	5.67 ± 2.37	0.077	0.53
TNF-a (pg/mL)	5.61 ± 1.38	6.41 ± 1.60	0.059	0.51

IL-6, interleukin-6; PAI-1, plasminogen activator inhibitor-1; TNF-a, tumor necrosis factor-a. Data are described by means ± SD. Significant (*p* < 0.05) difference between RGs and UCs *. ES is small if ES > 0.1, moderate if ES > 0.3 or large if ES > 0.5.

**Table 3:** BAT characteristics data in the RGs and UCs female groups.

	RG (33)	UC (20)	*p* Value	ES
BAT	29.6 ± 14.9	28.0 ± 13.1	0.707	0.11
BAT factor 1	8.7 ± 6.4	7.2 ± 6.3	0.412	0.24
BAT factor 2	7.0 ± 4.6	6.9 ± 3.7	0.913	0.03
BAT factor 3	6.8 ± 4.0	7.2 ± 4.5	0.795	0.08
BAT rest factor	7.0 ± 2.0	7.1 ± 2.2	0.931	0.24

BAT, body attitude test. Mean ± SD are shown. ES is small if ES > 0.1, moderate if ES > 0.3 or large if ES > 0.5.

**Table 4:** Spearman correlation coefficients between BAT and different body composition variables, energy intake and blood biomarkers in the RG (*n* = 33) and UC (*n* = 20) groups.

	RG (33)	UC (20)
	BAT	BAT
Body mass (kg)	0.190	0.402
BMI (kg/m^2^)	0.220	0.478 *
TB FM% (%)	0.040	0.137
TB FM (kg)	0.065	0.325
TB LBM (kg)	0.222	0.360
Energy intake (kcal/day)	−0.221	−0.211
REE (kcal/day)	0.218	0.074
Ferritin (ng/mL)	−0.001	0.059
Insulin (ulU/mL)	0.270	0.279
Leptin (ng/mL)	0.169	0.187
IL-6 (pg/mL)	0.120	0.326
PAI-1 (ng/mL)	0.300	0.273
Resistin (ng/mL)	0.354 *	−0.007
TNF- a (pg/mL)	0.259	0.268

Statistically significant correlations are shown with * (*p* < 0.05).

**Table 5:** Results of the stepwise multiple regression analysis with BAT as the dependent variable and body height, body mass, BMI, TB FM %, TB FM, TB LBM, energy intake, training duration, ferritin, insulin, leptin, IL-6, PAI-1, resistin and TNF-a as independent variables in RGs and UCs.

Dependent	RG (33)	UC (20)
	BAT	BAT
Resistin	24.1	-
Res + BMI	40.8	-
Body mass	-	21.0

R^2^ × 100 is shown describing the percentage of variability of dependent variables the BAT can explain. All values are percentages.

## Data Availability

The data presented in this study are available on a request from the corresponding author for researchers who meet the criteria for access to confidential data.

## Abbreviations

BAT	body attitude test
BIP	body image perception
BMI	body mass index
BMR	basal metabolic rate
CV	coefficient of variation
DXA	dual-energy X-ray absorptiometry
FM	fat mass
FM %	fat mass percent
IL-6	interleukin-6
LBM	lean body mass
PAI- 1	plasminogen activator inhibitor-1
REE	resting energy expenditure
RGs	rhythmic gymnasts
SD	standard deviations
TB	total body
TNF-a	tumor necrosis factor-a
UCs	untrained controls
VCO_2_	carbon dioxide production
VO_2_	oxygen consumption

## Appendix A. Body Attitude Test

**Table A1:** BODY—ATTITUDE—TEST (BAT).

		Always	Usually	Often	Sometimes	Rarely	Never
1	When I compare myself with my peers’ bodies, I’m dissatisfied with my own						
2	My body appears to be a numb thing						
3	My hips seem too broad to me						
4	I feel comfortable within my own body						
5	I have a strong desire to be thinner						
6	I think my breasts are too large						
7	I’m inclined to hide my body (e.g., by loose clothing)						
8	When I look at myself in the mirror, I’m dissatisfied with my own body						
9	It’s easy for me to relax physically						
10	I think I’m too thick						
11	I feel my body as a burden						
12	My body appears as if it is not mine						
13	Some parts of my body look swollen						
14	My body is a threat for me						
15	My bodily appearance is very important to me						
16	My belly looks as if I am pregnant						
17	I feel tense in my body						
18	I envy others for their physical appearance						
19	There are things going on in my body that frighten me						
20	I am observing my appearance in the mirror						
